# Extracellular Vesicle-Loaded Oncogenic lncRNA NEAT1 from Adipose-Derived Mesenchymal Stem Cells Confers Gemcitabine Resistance in Pancreatic Cancer via miR-491-5p/Snail/SOCS3 Axis

**DOI:** 10.1155/2023/6510571

**Published:** 2023-01-30

**Authors:** Rongxiang Wu, Zhan Su, Le Zhao, Ruifeng Pei, Yiren Ding, Deqiang Li, Shuo Zhu, Lu Xu, Wei Zhao, Wuyuan Zhou

**Affiliations:** ^1^Jiangsu University, Zhenjiang 212013, China; ^2^Department of Hepatopancreatobiliary Surgery, Xuzhou Cancer Hospital, Xuzhou 221005, China

## Abstract

It is becoming increasingly evident that key mechanisms of mesenchymal stem cell (MSC) efficacy appear to associate with paracrine activities, and the delivery of cargos through extracellular vesicles (EVs) controls the mechanistic actions of MSCs. Thus, this study clarified a possible mechanism by which EV-encapsulated NEAT1 from adipose-derived mesenchymal stem cells (ADSCs) might mediate gemcitabine resistance in pancreatic cancer (PCa). Microarray profile suggested a differentially expressed lncRNA NEAT1 in PCa, and we determined its expression in PCa cells. NEAT1 was found to be upregulated in PCa. The binding affinity among NEAT1, miR-491-5p, and Snail was identified through bioinformatic analysis and experimental validation. NEAT1 competitively bound to miR-491-5p to elevate Snail expression and diminish SOCS3 expression. PCa cells were cocultured with EVs extracted from ADSCs, followed by assessment of malignant phenotypes, tumorigenesis, and gemcitabine resistance of PCa cells using gain- or loss-of-function experiments. ADSC-derived EVs carrying NEAT1 promoted PCa cell proliferation, migration, and gemcitabine resistance *in vitro* and enhanced tumorigenicity *in vivo* by inhibiting miR-491-5p and SOCS3 and upregulating Snail. Collectively, the findings from our study found a new potential strategy for gemcitabine resistance in PCa by illustrating the mechanistic insights of oncogenic ADSC-derived EVs-loaded NEAT1 via regulating the miR-491-5p/Snail/SOCS3 axis.

## 1. Introduction

Pancreatic cancer (PCa) remains a lethal malignancy [1] with a poor prognosis [2]. The incidence of PCa has increased gradually in recent years [3], with a 5-year survival rate (approximately 10% in the USA) [4] still remains unsatisfactory [5] due to difficulties in diagnosis, rapid progression, drug resistance, and lack of effective antitumor therapy [6]. The main treatments for PCa are surgery, chemotherapy, and radiotherapy [7–9]. Gemcitabine is the first-line drug for chemotherapy in PCa [10, 11]. However, chemoresistance largely limits the efficacy of gemcitabine in PCa [10, 12], which remains a significant clinical problem [13, 14]. Therefore, it is urgent to understand the molecular mechanism for gemcitabine resistance and identify new biomarkers for the treatment of PCa.

Extracellular vesicles (EVs) are membrane-bound particles secreted by many different cells [15] and involved in intercellular communication among different organs and tissues [16, 17]. Prior evidence has documented that EVs shuttled by adipose-derived mesenchymal stem cells (ADSCs) via paracrine secretion modulate proliferative and migratory potential of tumor cells, which allows for precise target and limits systemic adverse events [18, 19]. The clinical significance of EVs in PCa has also been reported [20]. A variety of existing studies have also confirmed that EVs play a vital role in the tumorigenesis, metastatic capacity, and chemoresistance of PCa [21, 22]. Moreover, EVs contain essential cargoes, including microRNAs (miRNA), noncoding RNAs (ncRNAs), lipids, and proteins, which can be transferred into recipient cells to modulate their phenotypes [23].

Of interest, EVs could carry long noncoding RNAs (lncRNAs) to participate in the development of PCa [24]. Of note, nuclear paraspeckle assembly transcript 1 (NEAT1) has been previously documented as an oncogenic lncRNA promoting tumorigenesis, metastatic potential, and chemoresistance in PCa [25], but this study fails to illuminate its mechanistic actions clearly. NEAT1 is a transcriptional mediator for multiple genes implicated in pancreatic carcinogenesis [26] via a competing endogenous RNA (ceRNA) mechanism interacting with miRNAs. Prior findings have suggested that NEAT1/miR-101-dependent release of DNA-PKcs augments the malignant behaviors of pancreatic ductal adenocarcinoma cells [27]. Whereas, how the effect of NEAT1 was delivered to cancer cells remains unclear. Besides, miR-491-5p was predicted as a sponge of lncRNA NEAT1 through the StarBase database in the current study. Although miR-491-5p has also confirmed to be implicated in the malignant features PCa under regulation of LINC00460 [28], its effects on gemcitabine resistance in PCa remains to be established.

Thus, we attempted to investigate the contributions and mechanisms of the lncRNA NEAT1 shuttled by ADSC-derived EVs in PCa progression and gemcitabine resistance by sequestering miR-491-5p.

## 2. Materials and Methods

### 2.1. In Silico Analysis

PCa-associated microarray datasets (GSE91035, GSE59357, and GSE32676) were retrieved from the GEO database. The GSE91035 microarray dataset included 8 normal pancreatic samples and 27 PCa samples to analyze PCa-related differentially expressed genes (DEGs). The GSE59357 microarray dataset contained 3 dasatinib-resistant PCa cell lines and 3 dasatinib-sensitive PCa cell lines to verify the expression of miR-491-5p in PCa-resistant cell lines. The GSE32676 microarray dataset included 7 normal pancreatic samples and 25 PCa cells to analyze DEGs in PCa cells.

DEGs were analyzed using the R language “limma” package based on the criteria of |log2FC| > 1 and *p* < 0.05, followed by heat map plotting by the “ggolot2” package. The Ualcan database was utilized to evaluate relationship between miR-491-5p expression and the prognosis of PCa patients.

### 2.2. Cell Lines

The normal human pancreatic cell line HPDE6c7 (CL0317, Hunan Fenghui Biotechnology Co., Ltd., Changsha, China), PCa cell lines PANC-1 (CRL-1469), CFPAC-1 (CRL-1918), BxPC-3 (CRL-1687), ASPC-1 (CRL-1682), PACA-2 (CRM-CRL-1420), SW1990 (CRL-2172), and HEK293T (CRL-11268) from ATCC (Manassas, VA) were cultured in medium supplemented with 10% FBS and 1% penicillin/streptomycin with 5% CO_2_ at 37°C. McCoy's 5a (Sigma-Aldrich, St Louis MO) medium was used for the following: HPDE6c7 cell culture; IMDM (Gibco) for CFPAC-1 cell culture; DMEM for PANC-1, PACA-2, and HEK293T cells; RPMI-1640 medium for BxPC-3 and ASPC-1 cells; Leibovitz's L-15 (Gibco) medium for SW1990 cells.

### 2.3. Isolation and Identification of ADSCs

Human ADSCs (Zhong Qiao Xin Zhou Biotechnology Co., Ltd., Shanghai, China) were incubated in DMEM containing 10% FBS, 1% double antibody, and 2 mM L-glutamine (Sangon, Shanghai, China), which was maintained with 5% CO_2_ at 37°C for 48 h. When the confluency of ADSCs reached 80% to 90%, ADSCs were treated with trypsin and prepared for passage.

Cell morphology was observed under the light microscope (Olympus Corporation, Tokyo, Japan). ADSCs were then cultured in OriCell™ ADSC osteogenic (Zhong Qiao Xin Zhou Biotechnology), adipogenic (Procell, Wuhan, China), and chondrogenic differentiation (Gibco) medium, respectively. The stainings of alizarin red S (Sigma-Aldrich) [29], oil red O (Sigma-Aldrich) [30], and alcian blue (Sigma-Aldrich) [31] were performed to identify osteogenic, adipogenic, and chondrogenic differentiation, respectively.

Expression of surface markers of MSCs was detected by flow cytometry using the FACSVerse instrument (BD Biosciences, Franklin Lakes, NJ) and quantified using FlowJo software [32]. Cells were probed with antibodies (Abcam): CD105 (ab2529), CD73 (ab202122), CD90 (ab23894), CD31 (ab9498), CD19 (ab134114), or HLA-DR (ab92511). The goat anti-mouse IgG antibody (ab96899) served as isotype control.

### 2.4. Cell Transduction

The lipofectamine 2000 kit- (11668500, Invitrogen, CA) based cell transduction was performed upon reaching 70% cell confluence, and lentiviral infection was performed according to the instructions [33].

ADSCs were transduced with lentivirus (GenePharma Ltd., Shanghai, China) containing short hairpin RNA- (sh) negative control (NC, 5 g/ml) or sh-NEAT1 (5 g/ml). SW1990 cells were treated with mimic (20 nM, RiboBio Co., Ltd., Guangzhou, China) or inhibitor (50 nM, RiboBio Co., Ltd., Guangzhou, China) of miR-491-5p or NC, as well as lentiviral NEAT1, Vector (5 *μ*g/ml), SOCS3 (5 *μ*g/ml), or Snail (5 *μ*g/ml).

### 2.5. Extraction and Identification of EVs

EVs were isolated from ADSCs using the total EV kit (Invitrogen). The ADSCs were incubated in the FBS-free medium for 48 h to collect supernatant. The supernatant was centrifuged at 2000 g at 4°C for 30 min to remove cells and debris. Next, the supernatant was centrifuged at a 100 kDa ultrafiltration device at 4000 g at room temperature for 15 min to partially enrich EVs, which was mixed with 0.5-fold volume of total EV isolation reagent. The mixture was incubated at 4°C overnight and centrifuged at 10000 g at 4°C for 60 min the next day. Precipitates were the isolated EVs, resuspended in PBS, or stored at -80°C.

In addition, the protein levels of such EV-related markers as CD9 (ab236630, 1 : 1000, Abcam), CD63 (ab216130, 1 : 1000, Abcam), ALIX (ab88388, 1 : 1000, Abcam), and calnexin (ab133615, 1 : 5000, Abcam) were measured by Western blot analysis.

The morphological characteristics of EVs were observed under the transmission electron microscope (TEM) (Hitachi H7650, Tokyo, Japan) [34]. Particle size distribution of EVs was evaluated under a NanoSight nanoparticle tracking analyzer (NTA, Malvern, Marvin, UK) [35].

### 2.6. Uptake of EVs by PCa Cells

To determine the internalization of EVs by PCa cells, the isolated EVs were labeled with the lipophilic dye PKH67 (PKH67 Green Fluorescent Cell Linker Mini Kit, MINI67, Sigma-Aldrich) [36]. PCa cells (3 × 10^4^) were cocultured with PKH67-labeled EVs for 12 h. Cells were fixed in 4% paraformaldehyde. Uptake was then visualized by fluorescence microscope. ADSCs were cocultured with overexpressing Cy3-labeled NEAT1, and SW1990 cells were treated with the EV release inhibitor GW4869 (Sigma-Aldrich). After coculture for 12 h, ADSCs were fixed in 4% paraformaldehyde. NEAT1 uptake was then visualized by fluorescence microscope. Cytoskeleton was stained with phalloidin (Sigma), and nucleus was stained with DAPI (Sigma-Aldrich). ADSC-conditioned medium (CM) was treated with RNase A. Subsequently, NEAT1 expression was measured by reverse transcription quantitative polymerase chain reaction (RT-qPCR) to substantiate that NEAT1 was encapsulated with EVs. To exclude that NEAT1 expression was endogenously induced, PCa cells were treated with the RNA polymerase inhibitor actinomycin D (0.1 *μ*M, Sigma-Aldrich), followed by RT-qPCR detection of NEAT1 expression.

### 2.7. Coculture of ADSCs with PCa Cells

PCa cell line SW1990 and ADSCs were spread in chambers (0.4 *μ*m) at a ratio of 3 : 1, with ADSCs (1.2 × 10^5^) seeded in the upper chamber and SW1990 cells (0.4 × 10^5^) in the lower chamber. The coculture chambers were placed in the 6-well plates, with the upper chamber containing 10% serum and the lower chamber containing 15% serum, followed by coculture for 4 ~ 5 days. Medium was renewed once every 1 ~ 2 days. The cells in the upper chamber were treated with sphingomyelinase inhibitor GW4869 (10 *μ*M, Sigma-Aldrich). Cytoskeleton was labeled with phalloidin (AAT Bioquest, CA) to observe the NEAT1 position.

### 2.8. RT-qPCR

Total RNA of tissues and cells was extracted using Trizol (Thermo Fisher Scientific). The cDNA of mRNA and lncRNA was obtained using the reverse transcription kit (Takara, Tokyo, Japan), while that of miRNA containing PolyA tail was acquired by PolyA tailing test kit (Sangon). The samples were subjected to RT-qPCR based on the 2^−ΔΔCt^ method using the SYBR^®^ Premix Ex Taq™ II kit (Takara) on a real-time PCR instrument (ABI 7500, ABI, Foster City, CA), as normalized to U6 or GAPDH. The primer sequences are shown in Table S1.

### 2.9. Western Blot Analysis

Tissue or cell samples were lysed in RIPA lysis buffer, followed by concentration quantification using a BCA kit (Thermo Fisher Scientific). Protein lysates were loaded on SDS-PAGE and electrotransferred onto PVDF membranes. After being blocked with 5% skim milk, membranes were probed with primary antibodies overnight at 4°C, followed by incubation with secondary antibody goat anti-rabbit IgG (1 : 10000, ab205718, Abcam) at room temperature for 2 h. ECL reagents (MedChemExpress, Shanghai, China) were applied for visualization of immunoreactive bands. Protein band exposure was performed on an Image Quant LAS 4000C Gel Imager (General Electric Company, MA) and analyzed by Quantity One v4.6.2 software. The primary antibodies are as follows: Snail (1 : 1000, #3879, Cell Signaling Technology, Beverly, MA), SOCS3 (1 : 1000, ab16030, Abcam), and GAPDH (1 : 2500, ab9485, Abcam; loading control).

### 2.10. Colony Formation and Transwell Assays

SW1990 cells were plated in 6-well plates (500 cells/well), followed by incubation for 2-3 weeks. Cells were fixed with 4% formaldehyde and stained with crystal violet. A number of colonies with more than 10 cells were counted under the microscope with colony formation rate = (colony number/seeded cell number) × 100% [37].

Transwell chambers (8 *μ*m, Corning, NY) were applied for cell migration evaluation [38]. SW1990 cells resuspended with 200 *μ*l serum-free medium were plated in the upper chamber, with 10% FBS medium added in lower chamber. After 24 h, cells were fixed in methanol (Guangzhou Chemical Reagent Factory, Guangzhou, China) for 30 min. The crystal violet- (0.1%) stained cells were counted under an inverted microscope (Caikon Optical Instrument Co. Ltd., Shanghai, China).

### 2.11. MTS Assay

SW1990 cells were first cultured under normal culture conditions. Cell proliferation was analyzed using the MTS Cell Proliferation Kit (ab197010, Abcam). The cells were treated with 10 *μ*M gemcitabine (GEM; LY-188011, MedChemExpress) for 5 days to detect the sensitivity of SW1990 cells with DMSO as a NC.

### 2.12. Animal Experiments

Sixty NOD/SCID mice (4-6 weeks old) were purchased from Shanghai SLAC Laboratory Animal Co., Ltd. (Shanghai, China) and maintained under pathogen-free conditions with humidity of 50-65% at 26-28°C. All animal procedures were approved by the Institutional Animal Care and Use Committee of Xuzhou Cancer Hospital (2021-10-023-D07).

A subcutaneous xenograft model was established by subcutaneously injecting with PCa cells SW1990 (1 × 10^6^ cells; 200 *μ*l) into the axillary of mice. The tumor volumes were measured as (width^2^ × length)/2. When the tumor volume reached 50 mm^3^, mice were randomly grouped (*n* = 6) for further experiments. EVs (10 *μ*g) resuspended in 20 *μ*l PBS were injected into the center of the xenograft tumor every three days. The same volume of PBS was used as a control. Mice were euthanized on the 27^th^ day, and tumors were removed and photographed.

Mice were injected with PBS, EVs, EVs+sh-NEAT1, Vector+NC mimic EVs, NEAT1+NC mimic EVs, NEAT1+miR-491-5p mimic EVs, PBS+Vector, EVs+Vector, or EVs+SOCS3 via tail vein.

### 2.13. Fluorescent In Situ Hybridization (FISH)

The Cy3-labeled NEAT1 and FITC-labeled miR-491-5p probes were purchased from RiboBio. Cells were fixed in 4% formaldehyde and permeabilized with 0.5% Triton X-100. Cells were then hybridized to both Cy3- and FITC-labeled probes [39]. The nuclei were counterstained with DAPI, with images acquired under a confocal microscope (LEXT™ OLS5100, Olympus, Tokyo, Japan).

### 2.14. Dual-Luciferase Reporter Gene Assay

The binding sites were predicted through StarBase analysis, which was further validated by dual-luciferase reporter gene assay [40]. The dual-luciferase reporter vectors of NEAT1 and Snail and mutant (MUT) mutated with miR-491-5p binding sites, pGLO-NEAT1 wild-type (WT) (uuggcccaacacaUUCCCCACc) and pGLO-NEAT1 MUT (uuggcccaacacaAAGGGGUGc), pGLO-Snail WT (cagCAGGAAGGACCCCACa), and pGLO-Snail MUT (cagGU-CCUUCCAGGGGUGa) were constructed, respectively. The reporter plasmids were cotransfected with miR-491-5p mimic and NC mimic into HEK-293T and SW1990 cells, respectively. The Dual-Luciferase® Reporter Assay System (E1910, Promega, Madison, WI) was utilized to measure luciferase activity as normalized to Renilla luciferase activity.

### 2.15. RNA Immunoprecipitation (RIP) Assay

SW1990 cells were lysed with RIPA lysis buffer, and RIP assay was performed with Magna RIP™ RNA-Binding Protein Immunoprecipitation Kit (17-704, Millipore) [34]. The Argonaute 2 antibody (anti-Ago2, 67934-1-lg, 1 : 1000) and the normal IgG antibody (anti-IgG, 30000-0-AP, 1 : 1000, Proteintech Group Inc., IL) were incubated with magnetic beads (370-12D, Invitrogen) at 4°C for 1 h. Then, the cell lysates were incubated with the magnetic beads at 4°C overnight. After purification, the enrichment of NEAT1 and miR-491-5p was determined by RT-qPCR.

### 2.16. RNA Pull-Down Assay

Cells were lysed with RIPA lysis buffer and incubated with biotin-labeled Bio-NC and biotin-labeled NEAT1 (Bio-NEAT1) at 37°C for 1 h and then with streptavidin agarose beads (SA10004, Invitrogen) at 37°C for 1 h. The eluate was collected to measure the miR-491-5p expression using RT-qPCR.

### 2.17. Statistical Analysis

All data were presented as mean ± standard deviation and analyzed by SPSS 21.0 (IBM Corp., Armonk, NY). The difference was statistically significant at *p* < 0.05. The unpaired *t*-test for two-group comparison and one-way analysis of variance (ANOVA) with Tukey's post hoc test for multigroup comparison was included. Data at different time points were analyzed by repeated measurement ANOVA, followed by Tukey's post hoc test.

## 3. Results

### 3.1. NEAT1 Is Highly Expressed in Both PCa Tissues and Cells

**Figure 1:**
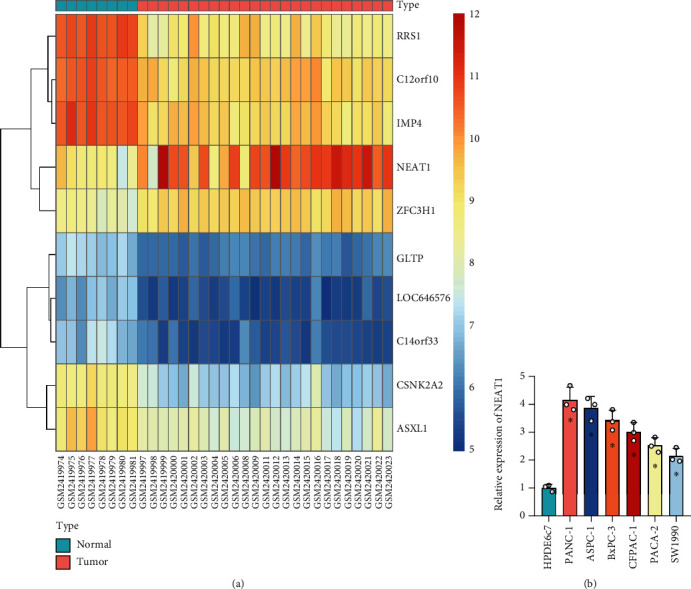
Target genes and cell lines were screened based on bioinformatics analysis and *in vitro* cell experiments. (a) Unsupervised cluster heat map of differentially expressed lncRNAs in microarray dataset GSE91035 (8 normal pancreatic samples and 27 PCa samples). (b) NEAT1 expression in PCa cell lines (PANC-1, CFPAC-1, BxPC-3, ASPC-1, PACA-2, and SW1990) and normal human pancreatic cell line HPDE6c7 determined by RT-qPCR. ^∗^*p* < 0.05 vs. HPDE6c7 cells. All data were presented as mean ± standard deviation. Comparison of data among multiple groups was tested by one-way analysis of variance, followed by Tukey's post hoc test. Cell experiments were independently repeated three times.

To investigate the role of lncRNAs in PCa, the microarray dataset GSE91035 was analyzed, and the heat map of the top 10 differentially expressed lncRNAs was drawn (Figure 1(a)) with NEAT1 presenting the most significant difference. Therefore, NEAT1 was selected as the target gene for subsequent experiments.

It was also found that NEAT1 expression was higher in PCa cell lines (PANC-1, CFPAC-1, BxPC-3, ASPC-1, PACA-2, and SW1990) than that in normal human pancreatic cell line HPDE6c7. The NEAT1 expression in PANC-1 cells was the highest, and it was relatively low in SW1990 cells (Figure 1(b)).

Thus, SW1990 cells were chosen for the following experiments.

### 3.2. ADSC-Derived EVs Could Deliver NEAT1 into PCa Cells

In this study, ADSCs were characterized by flow cytometry, which showed high expression of positive markers CD73, CD90, and CD105 and poor expression of negative markers CD31, CD19, and HLA-DR in ADSCs (Figure S1A). *In vitro* induction experiments also confirmed that ADSCs had the abilities of adipogenic, osteogenic, and chondrogenic differentiation (Figure S1B).

The ADSCs were further cultured to extract EVs. Typical bilayer and cup structures of EVs could be observed under the TEM (Figure S1C). NTA exhibited that the average diameter of ADSC-secreted EVs was about 100 nm (Figure S1D), which was within the typical EV size range (30 nm-150 nm). Besides, Western blot analysis revealed that EV marker proteins (CD9, ALIX, and CD63) were significantly expressed in EVs, and EV negative marker calnexin was not expressed (Figure S1E).

**Figure 2:**
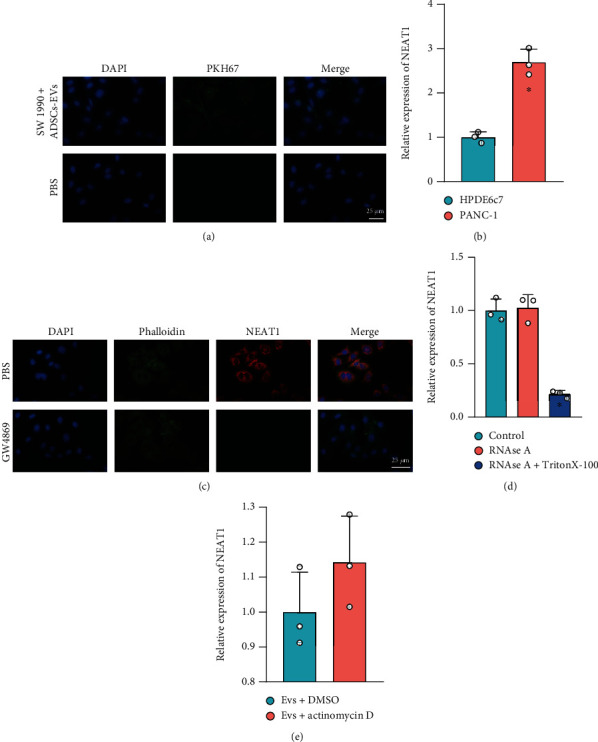
NEAT1 is delivered into PCa cells via ADSC-EVs. (a) Internalization of PKH67-labeled EVs by SW1990 cells under the fluorescence microscope (scale bar: 25 *μ*m). (b) NEAT1 expression in SW1990 cells cocultured with ADSC-EVs determined by RT-qPCR. (c) Delivery of NEAT1 by SW1990 cells cocultured with GW4869-treated ADSCs with Cy3-labeled NEAT1 overexpression (scale bar: 25 *μ*m). (d) NEAT1 expression in RNase A-treated ADSC-CM determined by RT-qPCR. (e) NEAT1 expression in SW1990 cells cocultured with actinomycin D determined by RT-qPCR. ^∗^*p* < 0.05 vs. SW1990 cells treated with PBS, ADSCs, or SW1990 cells cocultured with EVs+DMSO. All data were presented as mean ± standard deviation. Comparisons of data between two groups were analyzed by unpaired *t*-test, and comparison of data among multiple groups was tested by one-way analysis of variance, followed by Tukey's post hoc test. Cell experiments were independently repeated three times.

To further investigate the effect of ADSC-derived EVs on NEAT1 expression in PCa cells, PKH67-labeled EVs were cocultured with SW1990 cells for 12 h. The significant green fluorescence occurred in the cytoplasm of SW1990 cells (Figure 2(a)), which indicated that SW1990 cells could internalize EVs, and NEAT1 expression was upregulated in SW1990 cells cocultured with EVs (Figure 2(b)).

Cy3-labeled NEAT1 was overexpressed in ADSCs, which were cocultured with SW1990 cells to further identify whether NEAT1 was delivered by EVs into SW1990 cells. Significantly lower red fluorescence was observed in GW4869-treated SW1990 cells than that in untreated groups (Figure 2(c)), indicating that NEAT1 entered into recipient cells with ADSC-derived EVs as a carrier.

Next, it was witnessed that NEAT1 expression in the ADSCs in RNase A-treated CM was basically the same as in the control group, while NEAT1 was barely detectable in the ADSCs treated with RNase A+Triton X-100 (Figure 2(d)), suggesting that NEAT1 was stored in EVs.

To further exclude that NEAT1 expression was endogenously induced, SW1990 cells were treated with actinomycin D. RT-qPCR found no significant difference in NEAT1 expression between SW1990 cells cocultured with EVs+actinomycin D and SW1990 cells cocultured with EVs+DMSO (Figure 2(e)), which showed that NEAT1 expression was not endogenously induced.

These findings demonstrated that the ADSC-derived EVs could deliver NEAT1 into the PCa cells, thus promoting NEAT1 expression in the PCa cells.

### 3.3. ADSC-Derived EV-Encapsulated NEAT1 Promotes PCa Cell Proliferation, Migration, and *In Vivo* Tumorigenicity

**Figure 3:**
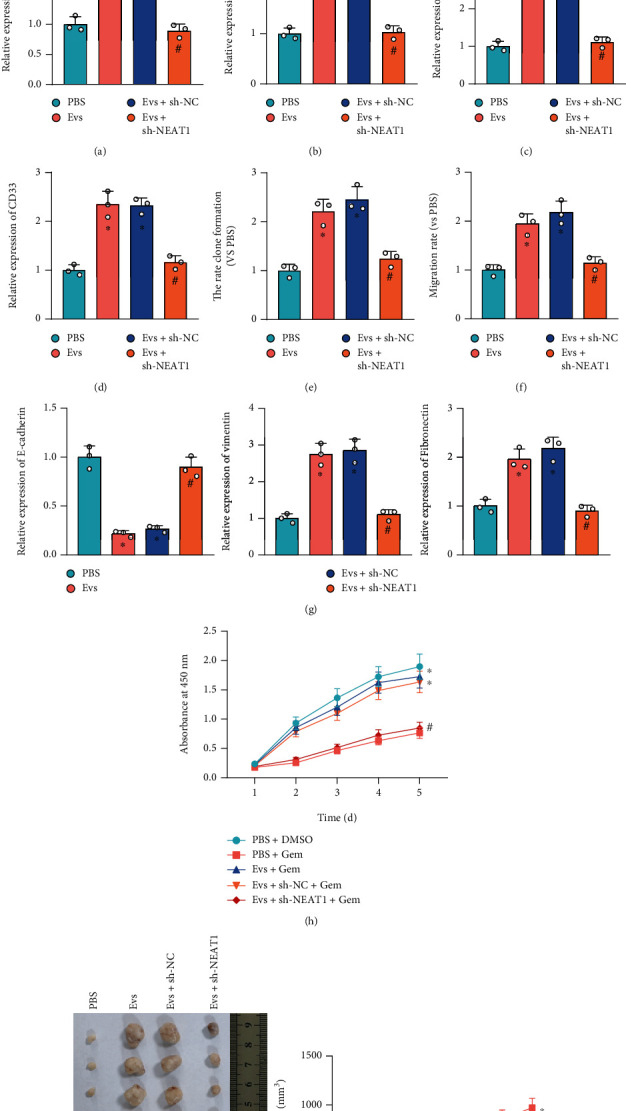
Effects of NEAT1 shuttled by ADSC-derived EVs on the biological properties of PCa cells. (a) Silencing efficiency of sh-NEAT1 in ADSCs detected by RT-qPCR. (b) NEAT1 expression in EVs from ADSCs transduced with sh-NEAT1-1 determined by RT-qPCR. (c) NEAT1 expression in SW1990 cells cocultured with EVs from sh-NEAT1-treated ADSCs measured by RT-qPCR. (d) The expression of stemness genes (CD33, OCT-4, NANOG, and CD90) in SW1990 cells cocultured with EVs from sh-NEAT1-treated ADSCs measured by RT-qPCR. (e) Clonogenic potential of SW1990 cells cocultured with EVs from sh-NEAT1-treated ADSCs detected by colony formation assay. (f) Migration of SW1990 cells cocultured with EVs from sh-NEAT1-treated ADSCs detected by Transwell assay. (g) Expression of EMT-related genes (E-cadherin, vimentin, and fibronectin) of SW1990 cells cocultured with EVs from sh-NEAT1-treated ADSCs measured by RT-qPCR. (h) Resistance of SW1990 cells cocultured with EVs from sh-NEAT1-treated ADSCs to gemcitabine detected by MTS assay. (i) Tumorigenicity of SW1990 cells in mice injected with SW1990 cells cocultured with EVs from sh-NEAT1-treated ADSCs (*n* = 6). ^∗^*p* < 0.05 vs. SW1990 cells transduced with sh-NC, SW1990 cells treated with PBS, or mice injected with SW1990 cells treated with PBS+DMSO or PBS+Gem; ^#^*p* < 0.05 vs. SW1990 cells cocultured with EVs from ADSCs transduced with sh-NC or mice injected with SW1990 cells treated with EVs+sh-NC+Gem. All data were presented as mean ± standard deviation. Comparisons of data between two groups were analyzed by unpaired *t*-test, and comparisons of data among multiple groups were tested by one-way analysis of variance (ANOVA), followed by Tukey's post hoc test. Data at different time points were analyzed by repeated measurement ANOVA, followed by Tukey's post hoc test. Cell experiments were independently repeated three times.

This study moved to further study the effect of EVs carrying NEAT1 in PCa cells. NEAT1 was knocked down by shRNA in ADSCs, and sh-NEAT1-1 exhibited optimal silencing efficiency, and sh-NEAT1-1 was selected for subsequent experiments (Figure 3(a)).

Moreover, SW1990 cells were cocultured with EVs from sh-NEAT1-treated ADSCs (Figure 3(b)). RT-qPCR showed that NEAT1 expression was increased in SW1990 cells cocultured with EVs from ADSCs, while NEAT1 expression was decreased in SW1990 cells cocultured with EVs from sh-NEAT1-treated ADSCs (Figure 3(c)).

SW1990 cells cocultured with ADSC-EVs presented elevated expression of stemness genes (CD33, OCT-4, NANOG, and CD90) (Figure 3(d)), promoted clonogenic potential (Figure 3(e), Figure S2A) and migration (Figure 3(f), Figure S2B), decreased E-cadherin level, increased levels of vimentin and fibronectin (Figure 3(g)), and enhanced SW1990 cell resistance to gemcitabine (Figure 3(h)), while these effects were reversed by further downregulation of NEAT1. In addition, treatment with ADSC-EVs promoted SW1990 cell proliferation and *in vivo* tumorigenicity, while further silencing of NEAT1 suppressed SW1990 cell proliferation and *in vivo* tumorigenicity (Figure 3(i)).

The results confirmed that ADSC-derived EVs carrying NEAT1 induced the proliferation, migration, and *in vivo* tumorigenicity of PCa cells.

### 3.4. NEAT1 Competitively Binds to miR-491-5p in PCa Cells

**Figure 4:**
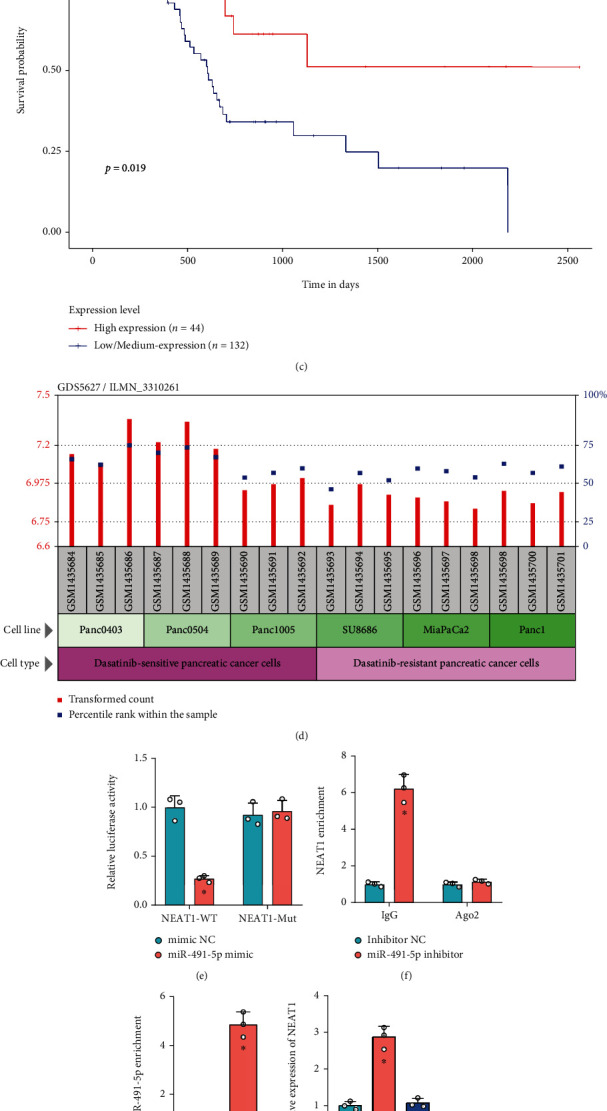
Verification of the binding between NEAT1 and miR-491-5p in PCa cells. (a) The binding site between NEAT1 and miR-491-5p in PCa cells predicted by the StarBase database. (b) The colocalization of NEAT1 and miR-491-5p detected by FISH (scale bar: 25 *μ*m). (c) The relationship between miR-491-5p expression and the prognosis of PCa patients (44 patients with high miR-491-5p expression and 132 patients with low miR-491-5p expression) analyzed by Ualcan database. (d) miR-491-5p expression in dasatinib-sensitive PCa cell lines and dasatinib-resistant PCa cell lines analyzed in GSE59357 (*n* = 3). (e) Luciferase activity of NEAT1-WT/MUT analyzed by dual-luciferase reporter gene assay. (f) Enrichment of miR-491-5p by NEAT1 in SW1990 cells treated with anti-Ago2 or anti-IgG detected by RIP. (g) RNA pull-down assay for a biotinylated NEAT1 and an NC probe, miR-491-5p enrichment detected by RT-qPCR. (h) NEAT1 expression in SW1990 cells treated with overexpression vector or shRNA-mediated NEAT1 measured by RT-qPCR. (i) miR-491-5p expression in SW1990 cells treated with overexpression vector or shRNA-mediated NEAT1 measured by RT-qPCR. (j) miR-491-5p expression in SW1990 cells cocultured with EVs from sh-NEAT1-treated ADSCs measured by RT-qPCR. ^∗^*p* < 0.05 vs. SW1990 cells transduced with mimic NC or inhibitor NC, SW1990 cells treated with Bio-NC-probe, Vector, or PBS; ^#^*p* < 0.05 vs. SW1990 cells transduced with sh-NC or SW1990 cells cocultured with EVs from ADSCs transduced with sh-NC. All data were presented as mean ± standard deviation. Comparisons of data between two groups were analyzed by unpaired *t*-test, and comparison of data among multiple groups was tested by one-way analysis of variance, followed by Tukey's post hoc test. Cell experiments were independently repeated three times.

StarBase database analysis predicted that NEAT1 could bind to miR-491-5p (Figure 4(a)). In addition, FISH verified that NEAT1 and miR-491-5p were colocalized in SW1990 cells (Figure 4(b)). The Ualcan database also found that high expression of miR-491-5p indicated the better prognosis of patients with PCa (Figure 4(c)). In addition, analysis of microarray dataset GSE59357 found that miR-491-5p expression in dasatinib-sensitive PCa cells was higher than that in dasatinib-resistant PCa cells (Figure 4(d)), which suggested that miR-491-5p expression was related to the malignant progression and drug resistance of PCa.

The binding of NEAT1 and miR-491-5p was verified by dual-luciferase reporter gene assay, which displayed that the luciferase activity of NEAT1-WT in HEK293T cells was inhibited by miR-491-5p mimic, while no obvious difference was found in NEAT1-MUT (Figure 4(e)). RIP analysis showed that NEAT1 was detected in Ago2 immunoprecipitates in SW1990 cells transduced with NC inhibitor, but Ago2 complexes purified from SW1990 cells transduced with miR-491-5p inhibitor (Figure 4(f)). RNA pull-down assay presented that the biotin-labeled NEAT1 could enrich miR-491-5p (Figure 4(g)), indicating that miR-491-5p and NEAT1 could bind to each other.

In addition, overexpression of NEAT1 in SW1990 cells reduced miR-491-5p expression, while knockdown of NEAT1 upregulated miR-491-5p (Figures 4(h) and 4(i)). Similarly, miR-491-5p expression was reduced in SW1990 cells cocultured with EVs from ADSCs, while it was increased in SW1990 cells cocultured with EVs from sh-NEAT1-treated ADSCs (Figure 4(j)).

It could be concluded that NEAT1 could sponge miR-491-5p in PCa cells.

### 3.5. NEAT1 Promotes PCa Cell Proliferation, Migration, and Gemcitabine Resistance by Sponging miR-491-5p

**Figure 5:**
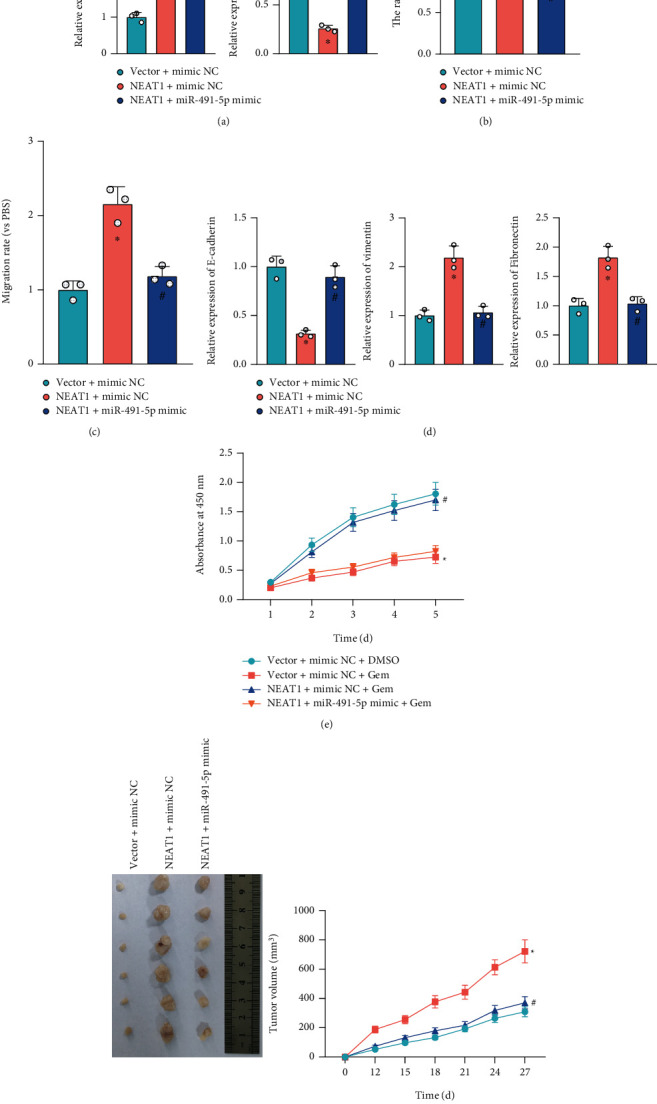
Effect of NEAT1 and miR-491-5p on PCa cell activities and gemcitabine resistance. SW1990 cells were transduced with overexpression vector-mediated NEAT1 alone or combined with miR-491-5p. (a) Expression of NEAT1 and miR-491-5p determined by RT-qPCR. (b) Clonogenic potential of SW1990 cells detected by colony formation assay. (c) Migration of SW1990 cells detected by Transwell assay. (d) Expression of EMT-related genes (E-cadherin, vimentin, and fibronectin) of SW1990 cells measured by RT-qPCR. (e) Resistance of SW1990 cells to gemcitabine detected by MTS assay. (f) Tumorigenicity of SW1990 cells in mice (*n* = 6). ^∗^*p* < 0.05 vs. SW1990 cells transduced with Vector+NC mimic or Vector+mimic NC+DMSO, or mice injected with SW1990 cells treated with Vector+NC mimic; ^#^*p* < 0.05 vs. SW1990 cells transduced with NEAT1+NC mimic or NEAT1+mimic NC+Gem, or mice injected with SW1990 cells treated with NEAT1+NC mimic. All data were presented as mean ± standard deviation. Comparisons of data among multiple groups were tested by one-way analysis of variance (ANOVA), followed by Tukey's post hoc test. Data at different time points were analyzed by repeated measurement ANOVA, followed by Tukey's post hoc test. Cell experiments were independently repeated three times.

To investigate whether NEAT1 affects PCa cell activities and gemcitabine resistance through miR-491-5p, SW1990 cells were introduced with overexpression vector-mediated NEAT1 alone or combined with miR-491-5p. RT-qPCR displayed that NEAT1 expression was elevated, while miR-491-5p expression was decreased in SW1990 cells transduced with NEAT1+NC mimic, while NEAT1 expression showed no evident difference, and miR-491-5p expression was increased in SW1990 cells transduced with NEAT1+miR-491-5p mimic (Figure 5(a)).

It was also found that SW1990 cells overexpressing NEAT1 showed promoted clonogenic potential, migration, EMT progression, and resistance to gemcitabine, while additional upregulation of miR-491-5p abolished these effects (Figures 5(b)–5(e), Figure S2C, D). In addition, *in vivo* animal experiments showed that overexpression of NEAT1 enhanced SW1990 cell proliferation and tumorigenicity *in vivo*, while they were counteracted by further overexpression of miR-491-5p (Figure 5(f)).

The obtained data suggested that NEAT1 could promote PCa cell proliferation, migration, and gemcitabine resistance by sponging miR-491-5p.

### 3.6. NEAT1 Regulates Snail and SOCS3 Expression by Competitively Binding to miR-491-5p in PCa Cells

**Figure 6:**
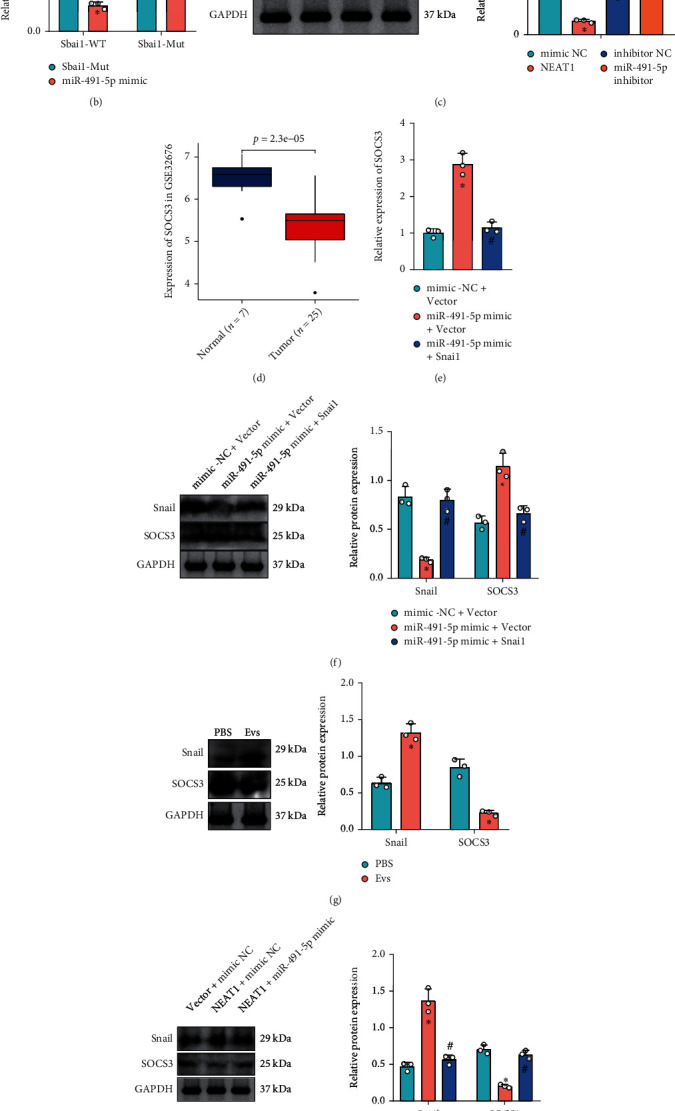
miR-491-5p regulates Snail/SOCS3 in PCa cells. (a) The binding site between miR-491-5p and Snail predicted by StarBase database. (b) The target relationship between miR-491-5p and Snail verified by dual-luciferase reporter gene assay. (c) Snail expression in SW1990 cells in response to miR-491-5p mimic alone or combined with Snail overexpression determined by Western blot analysis. (d) SOCS3 expression in the PCa-related microarray dataset GSE32676 (normal pancreatic tissues: *n* = 7; PCa tissues: *n* = 25). (e) SOCS3 mRNA level in SW1990 cells in response to miR-491-5p mimic alone or combined with Snail overexpression determined by RT-qPCR. (f) SOCS3 protein level in SW1990 cells determined by Western blot analysis. SW1990 cells were treated with EVs. (g) Protein levels of Snail and SOCS3 in SW1990 cells in response to EVs determined by Western blot analysis. (h) Protein levels of Snail and SOCS3 in SW1990 cells in response to overexpression of NEAT1 alone or combined with miR-491-5p determined by Western blot analysis. ^∗^*p* < 0.05 vs. SW1990 cells transduced with mimic NC, inhibitor NC, PBS, or Vector+NC mimic. ^#^*p* < 0.05 vs. SW1990 cells transduced with miR-491-5p mimic+Vector or NEAT1+NC mimic. All data were presented as mean ± standard deviation. Comparisons of data between two groups were analyzed by unpaired *t*-test, and comparison of data among multiple groups was tested by one-way analysis of variance, followed by Tukey's post hoc test. Cell experiments were independently repeated three times.

To further explore the mechanism of miR-491-5p in PCa, the StarBase database predicted that miR-491-5p could bind to Snail (Figure 6(a)). More importantly, dual-luciferase reporter gene assay exhibited that luciferase activity of Snail-WT in HEK293T cells was inhibited by miR-491-5p mimic, while no significant difference was found in Snail-MUT (Figure 6(b)).

Meanwhile, SW1990 cells were transduced with mimic or inhibitor of miR-491-5p. Western blot analysis (Figure 6(c)) exhibited that overexpression of miR-491-5p promoted Snail expression, while inhibition of miR-491-5p suppressed Snail expression, suggesting that miR-491-5p could specifically inhibit Snail expression. A previous study has indicated that Snail repressed the SOCS3 transcription [41]. Moreover, the analysis of the microarray dataset GSE32676 found that SOCS3 expression was significantly downregulated in PCa (Figure 6(d)). Therefore, it can be speculated that miR-491-5p could regulate SOCS3 expression through Snail.

*In vitro* experiments were further performed based on SW1990 cells. It was found that overexpression of miR-491-5p could elevate mRNA and protein levels of SOCS3, while further upregulation of Snail reduced mRNA and protein levels of SOCS3 (Figures 6(e) and 6(f)). Moreover, Western blot analysis presented that EVs or overexpression of NEAT1 promoted Snail expression and suppressed SOCS3 expression, while additional overexpression of miR-491-5p reduced Snail expression and elevated SOCS3 expression (Figures 6(g) and 6(h)).

These results confirmed that NEAT1 could downregulate miR-491-5p to promote Snail expression and inhibit SOCS3 expression in PCa cells.

### 3.7. NEAT1 Delivered by ADSC-Derived EVs Mediates the miR-491-5p/Snail/SOCS3 Axis to Promote PCa Malignant Phenotypes and Gemcitabine Resistance

**Figure 7:**
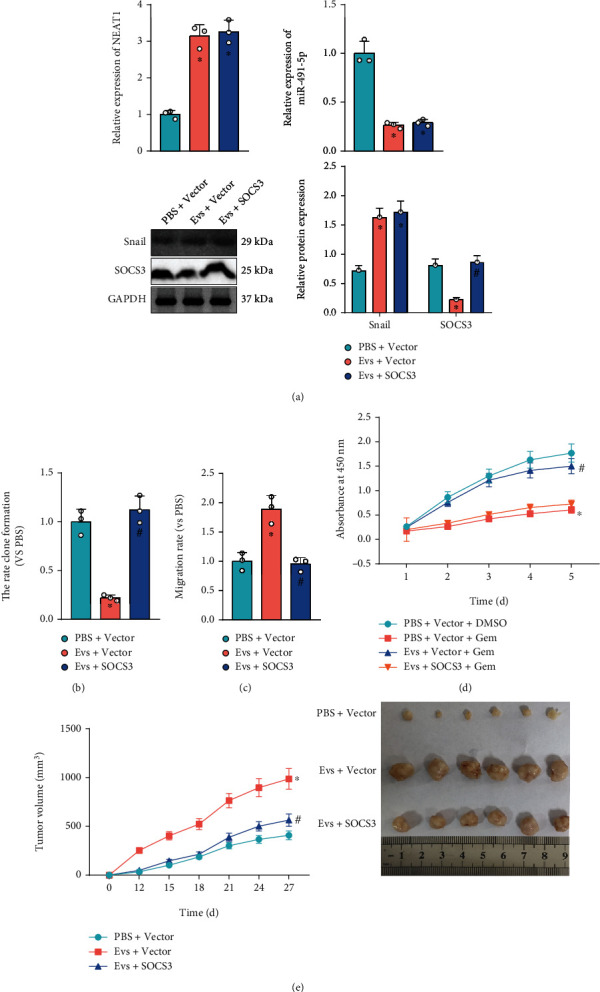
Effects of ADSC-EVs carrying NEAT1 on PCa progression and gemcitabine resistance via miR-491-5p/Snail/SOCS3 axis. SW1990 cells were treated with EVs alone or combined with overexpressed SOCS3. (a) Expression of NEAT1 and miR-491-5p determined by RT-qPCR and protein levels of Snail and SOCS3 in SW1990 cells determined by Western blot analysis. (b) Clonogenic potential of SW1990 cells detected by colony formation assay. (c) Migration of SW1990 cells detected by Transwell assay. (d) Resistance of SW1990 cells to gemcitabine detected by MTS assay. (e) Tumorigenicity of SW1990 cells cocultured with EVs alone or combined with SOCS3 overexpression treatment in mice (*n* = 6). ^∗^*p* < 0.05 vs. SW1990 cells treated with PBS+Vector+Gem or PBS +Vector, or mice injected with SW1990 cells treated with PBS+Vector; ^#^*p* < 0.05 vs. SW1990 cells treated with EVs+Vector or EVs+Vector+Gem, or mice injected with SW1990 cells treated with EVs+Vector. All data were presented as mean ± standard deviation. Comparison of data among multiple groups was tested by one-way analysis of variance (ANOVA), followed by Tukey's post hoc test. Data at different time points were analyzed by repeated measurement ANOVA, followed by Tukey's post hoc test. Cell experiments were independently repeated three times.

To determine the effect of EV-NEAT1/miR-491-5p/Snail/SOCS3 axis on PCa progression, SW1990 cells were treated with EVs alone or combined with overexpressed SOCS3. RT-qPCR and Western blot analysis indicated that NEAT1 and Snail were upregulated, and miR-491-5p and SOCS3 were downregulated in SW1990 cells treated with EVs, while SOCS3 expression was increased compared with SW1990 cells treated with EVs+SOCS3 (Figure 7(a)).

Moreover, it was also found that EV coculture strengthened SW1990 cell clonogenic potential, migration, EMT, and resistance to gemcitabine, while these effects were reversed by further treatment with upregulated SOCS3 (Figures 7(b)–7(d), Figure S2E, F). *In vivo* animal experiments also validated that treatment with EVs enhanced tumorigenicity of SW1990 cells *in vivo*, while further treatment with upregulated SOCS3 attenuated tumorigenicity of SW1990 cells (Figure 7(e)).

It can be concluded that ADSC-derived EVs accelerated PCa progression and gemcitabine resistance by mediating NEAT1/miR-491-5p/Snail/SOCS3 axis.

## 4. Discussion

Over the past decades, gemcitabine serves as the main drug for chemotherapy for PCa [42, 43]. However, gemcitabine resistance remains a tough obstacle in the treatment of PCa [44]. Moreover, gemcitabine resistance in PCa has also been reported to correlate with aberrant expression of lncRNAs [45, 46]. Although dysregulation of certain lncRNAs has been demonstrated in chemo-resistant cancers, the functional mechanisms of lncRNA NEAT1 shuttled by ADSC-EVs in PCa remain undetermined. In this study, we illuminated that the ADSC-EVs could deliver NEAT1 into PCa cells, and NEAT1 was upregulated in PCa, and subsequent gain- and loss-of-function experiments revealed that ADSC-EVs carrying NEAT1 promote PCa progression and gemcitabine resistance *in vitro* and *in vivo* by mediating the miR-491-5p/Snail/SOCS3 axis.

We observed that NEAT1 was highly expressed in both PCa tissues and cells. Consistently, a previous study has verified high expression of NEAT1 in PCa, which is closely associated with tumor progression and poor survival in patients with PCa [47], but this study failed to clarify the downstream mechanisms or how the NEAT1 action was realized in PCa cells. Our work furthered the understanding and suggested that ADSC-derived EVs could deliver NEAT1 into PCa cells to enhance cell proliferation, migration, gemcitabine resistance, and tumorigenicity both *in vitro* and *in vivo*. It has been demonstrated that EVs could transfer lncRNAs into PCa cells to affect the PCa initiation and progression [48]. Another study has also revealed that ADSC-derived exosomes could regulate tumor progression by mediating the malignant phenotypes [18]. Moreover, lncRNA NEAT1 could facilitate PCa cell growth, invasion, and migration [26]. It is interesting to note that downregulation of NEAT1 could inhibit the EMT process and improve sensitivity of PCa cells to gemcitabine by sponging miR-506-3p [49]. Our findings underscore the importance of NEAT1 delivered by ADSC-derived EVs in PCa progression and gemcitabine resistance.

In addition, it is well known that lncRNA NEAT1 acts as a ceRNA of miRNA to inhibit miRNA expression, eventually leading to the elevated expression of target gene [27]. The current study also exhibited that lncRNA NEAT1 could competitively bind to miR-491-5p to induce PCa cell proliferation, migration, and gemcitabine resistance. As previously described, miR-491-5p has been demonstrated to downregulate in PCa, and upregulated miR-491-5p could repress SW1990 cell growth [50, 51]. Another study has also validated that silencing of miR-491-5p by LINC00460 could accelerate the progression of PCa [28]. Evidence has been presented and demonstrated that miRNAs that regulate the gemcitabine resistance of PCa have been reported [52], while the roles of miR-491-5p in gemcitabine resistance of PCa still need to be further explored.

Furthermore, the obtained data in the present study indicated that miR-491-5p could target Snail in PCa. Snail, a member of the Snail family of transcription factors, is a critical regulator of EMT in PCa progression and PCa cell response to chemotherapy [53]. Recent evidence suggests that depleted Snail2 could inhibit tumorigenicity and resistance to gemcitabine in PCa [54]. A prior study has also suggested that Snail could affect the epigenetic silencing of SOCS3, which functions as a cytokine-inducible negative regulator of cytokine signaling [41]. A growing number of studies have confirmed that overexpression of SOCS3 could accelerate the PCa progression and gemcitabine resistance by inducing malignant features [55, 56]. Taken together, our data shuttled by demonstrating that NEAT1 ADSC-EVs functioned as a ceRNA to regulate the miR-491-5p/Snail/SOCS3 axis to facilitate PCa progression and gemcitabine resistance.

## 5. Conclusions

**Figure 8:**
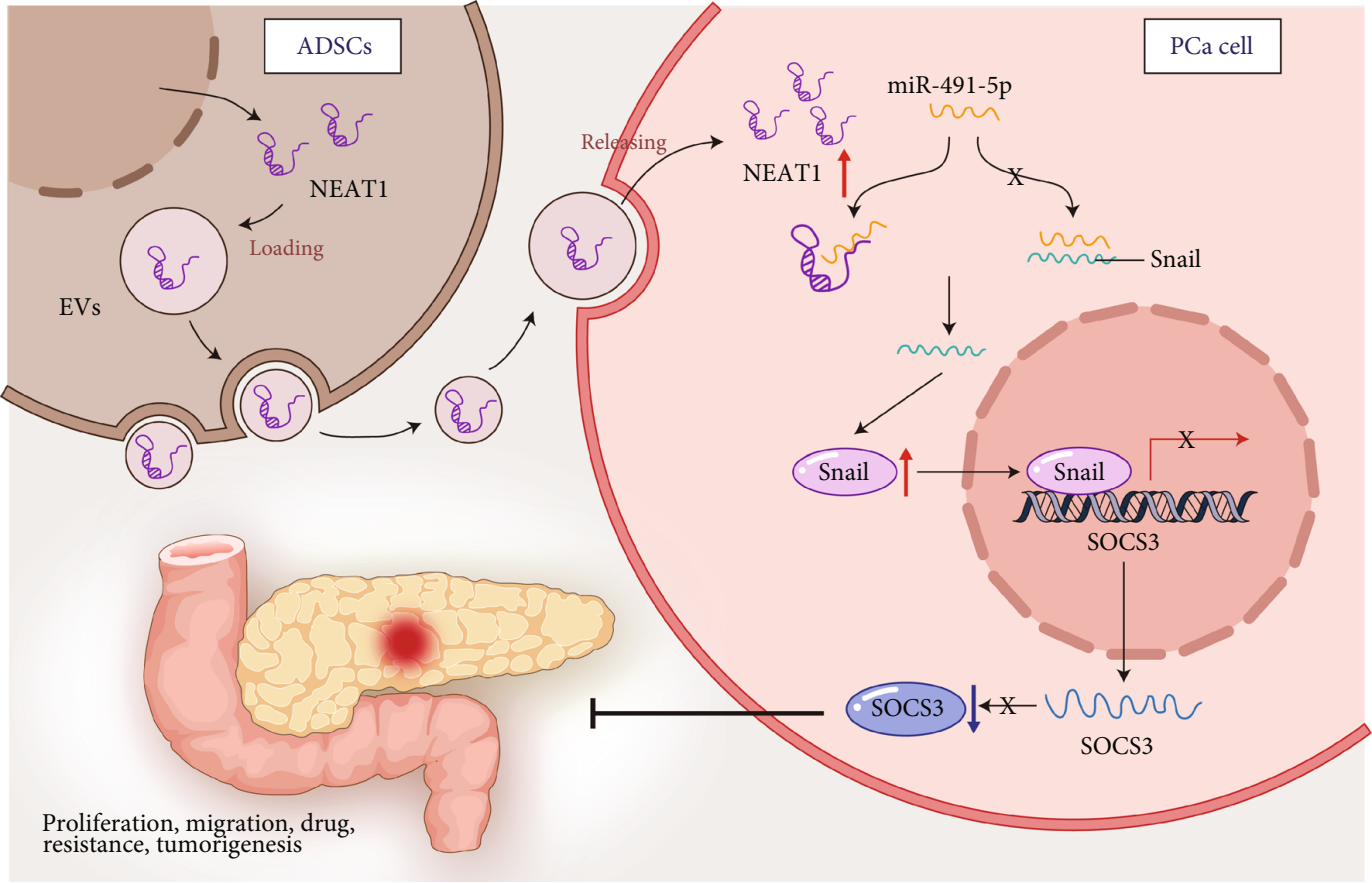
Molecular mechanism of NEAT1 shuttled by ADSC-derived EVs on malignant phenotypes and gemcitabine resistance of PCa via miR-491-5p/Snail/SOCS3 axis.

In summary, our study demonstrated that the transfer of NEAT1 via ADSC-EVs altered the expression of miR-491-5p, Snail, and SOCS3, all of which leads to the induction of a PCa progression and gemcitabine resistance (Figure 8). Our findings provide insights into the mechanistic actions of ADSC-EV-loaded NEAT1 in PCa chemo-resistance. However, whether the therapeutic target is applicable to human beings requires to be further verified. Additionally, the findings provided in this study are preliminary, indicating more studies in this area are required in the future.

## Data Availability

The datasets generated and/or analyzed during the current study are available from the corresponding authors on reasonable request.

## Supplementary Materials

Supplementary 1 Figure S1: identification of ADSCs and ADSC-EVs. (a) Expression of MSC markers detected by flow cytometry. (b) Detection of multipotent differentiation induction of ADSCs (scale bar: 50 *μ*m). (c) EV morphology observed under the TEM. (d) Size distribution of EVs detected by NTA. (e) Specific surface-labeled proteins of EVs detected using Western blot analysis. Cell experiments were independently repeated three times. Figure S2: representative images of clonogenic potential and migration of SW1990 cells. (a, b) Clonogenic potential (a) and migration (b) of SW1990 cells cocultured with EVs from sh-NEAT1-treated ADSCs detected by colony formation assay (a) and Transwell assay (b). (c, d) Clonogenic potential (c) and migration (d) of SW1990 cells in response to overexpression vector-mediated NEAT1 alone or combined with miR-491-5p detected by colony formation assay (c) and Transwell assay (d). (e, f) Clonogenic potential (e) and migration (f) of SW1990 cells in response to EVs alone or combined with overexpressed SOCS3 detected by colony formation assay (e) and Transwell assay (f).

Supplementary 2 Table S1: primer sequences of RT-qPCR. Note: RT-qPCR: reverse transcription quantitative polymerase chain reaction; NEAT1: nuclear-enriched abundant transcript 1; SNAI1: snail family transcriptional repressor 1; SOCS3: suppressor of cytokine signalling-3; 4-Oct: 4-octamer-binding transcription factor; GAPDH: glyceraldehyde-3-phosphate dehydrogenase.
