# Supplementary figures and images for: Extracellular Vesicle-Loaded Oncogenic lncRNA NEAT1 from Adipose-Derived Mesenchymal Stem Cells Confers Gemcitabine Resistance in Pancreatic Cancer via miR-491-5p/Snail/SOCS3 Axis
Source: Stem Cells Int. 2023 Jan 30;2023:6510571. doi: 10.1155/2023/6510571 (PMC9902843; doi:10.1155/2023/6510571)

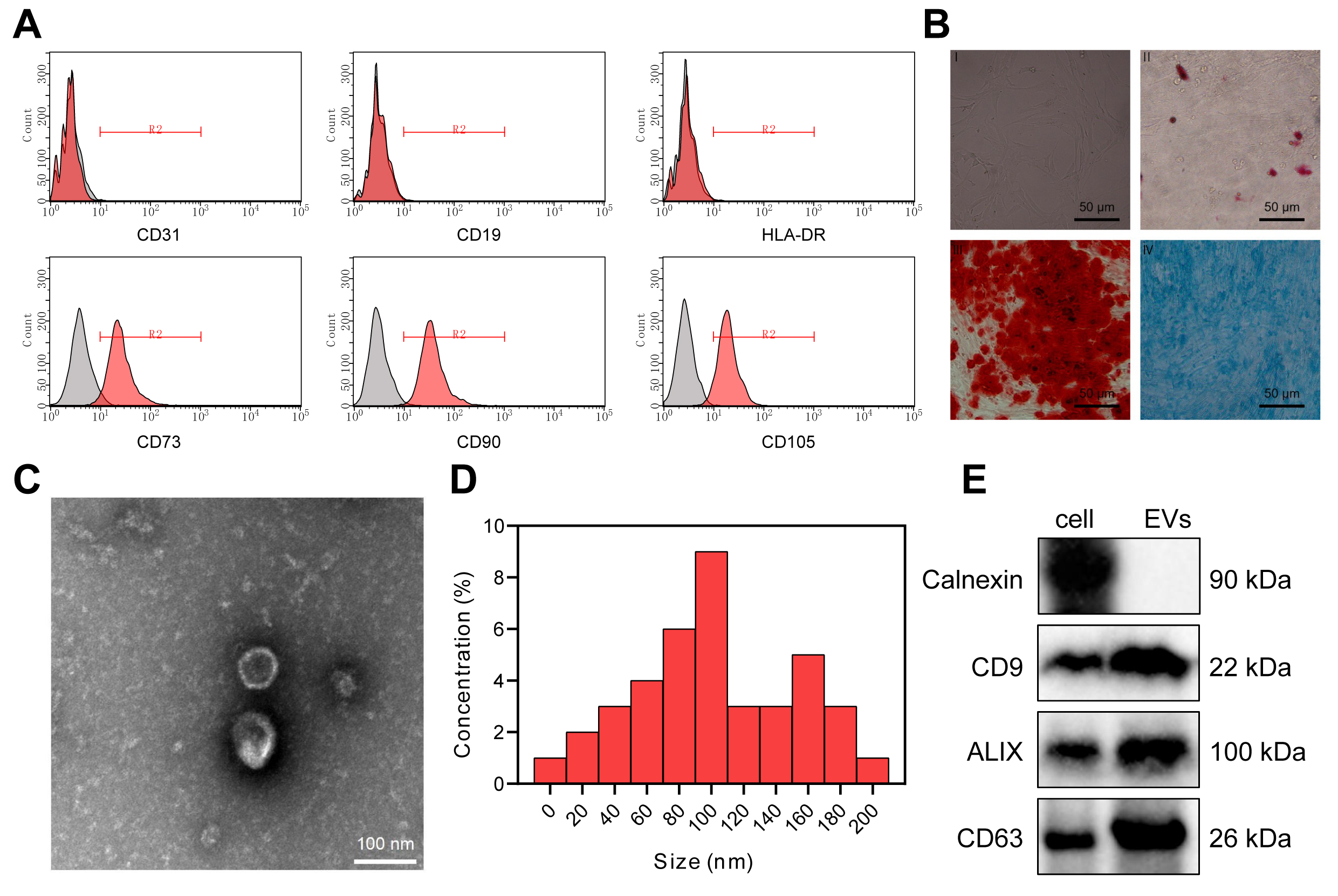

Supplement: Supplementary 1 — Figure S1: identification of ADSCs and ADSC-EVs. (a) Expression of MSC markers detected by flow cytometry. (b) Detection of multipotent differentiation induction of ADSCs (scale bar: 50 μm). (c) EV morphology observed under the TEM. (d) Size distribution of EVs detected by NTA. (e) Specific surface-labeled proteins of EVs detected using Western blot analysis. Cell experiments were independently repeated three times. Figure S2: representative images of clonogenic potential and migration of SW1990 cells. (a, b) Clonogenic potential (a) and migration (b) of SW1990 cells cocultured with EVs from sh-NEAT1-treated ADSCs detected by colony formation assay (a) and Transwell assay (b). (c, d) Clonogenic potential (c) and migration (d) of SW1990 cells in response to overexpression vector-mediated NEAT1 alone or combined with miR-491-5p detected by colony formation assay (c) and Transwell assay (d). (e, f) Clonogenic potential (e) and migration (f) of SW1990 cells in response to EVs alone or combined with overexpressed SOCS3 detected by colony formation assay (e) and Transwell assay (f). [file 6510571.f1.zip › 6510571.f1/New Figure S1.jpg]
