# Supplementary material for: Extracellular Vesicle-Loaded Oncogenic lncRNA NEAT1 from Adipose-Derived Mesenchymal Stem Cells Confers Gemcitabine Resistance in Pancreatic Cancer via miR-491-5p/Snail/SOCS3 Axis
Source: Stem Cells Int. 2023 Jan 30;2023:6510571. doi: 10.1155/2023/6510571 (PMC9902843; doi:10.1155/2023/6510571)
Supplement: Supplementary 2 — Table S1: primer sequences of RT-qPCR. Note: RT-qPCR: reverse transcription quantitative polymerase chain reaction; NEAT1: nuclear-enriched abundant transcript 1; SNAI1: snail family transcriptional repressor 1; SOCS3: suppressor of cytokine signalling-3; 4-Oct: 4-octamer-binding transcription factor; GAPDH: glyceraldehyde-3-phosphate dehydrogenase. [file 6510571.f2.docx]

**Table S1** Primer sequences of RT-qPCR

| Genes | Sequences (5'-3') |
| --- | --- |
| NEAT1 | Forward: 5'-GACCTCTCACCTACCCACCT-3' |
|  | Reverse: 5'-CTTGTACCCTCCCAGCGTTT-3' |
| miR-491-5p | Forward: 5'- AGTGGGGAACCCTTCCATGAGG-3' |
|  | Reverse: universal reverse primer |
| Snai1 | Forward: 5'-TCGGAAGCCTAACTACAGCGA-3' |
|  | Reverse: 5'-AGATGAGCATTGGCAGCGAG-3' |
| SOCS3 | Forward: 5'-GCAGGGAGGTGACGAGC-3' |
|  | Reverse: 5'-AAACTTGCTGTGGGTGACCA-3' |
| 4-Oct | Forward: 5'-GCCGCTGGCTTATAGAAGGT-3' |
|  | Reverse: 5'-ACATGGCATGCATACACACA-3' |
| NANOG | Forward: 5'-TTTGTGGGCCTGAAGAAAACT-3' |
|  | Reverse: 5'-AGGGCTGTCCTGAATAAGCAG-3' |
| CD33 | Forward: 5'-GGCCACTCCAAAAACCTGAC-3' |
|  | Reverse: 5'-GACAACCAGGAGAAGATCGGG-3' |
| CD90 | Forward: 5'-CTAGTGGACCAGAGCCTTCG-3' |
|  | Reverse: 5'-GCACGTGCTTCTTTGTCTCA-3' |
| E-cadherin | Forward: 5'-TGCCCAGAAAATGAAAAAGG-3' |
|  | Reverse: 5'-GTGTATGTGGCAATGCGTTC-3' |
| Vimentin | Forward: 5'-GAGAACTTTGCCGTTGAAGC-3' |
|  | Reverse: 5'-GCTTCCTGTAGGTGGCAATC-3' |
| Flbronectin | Forward: 5'-CAGTGGGAGACCTCGAGAAG-3' |
|  | Reverse: 5'-TCCCTCGGAACATCAGAAAC-3' |
| U6 | Forward: 5'-CTCGCTTCGGCAGCACA-3' |
|  | Reverse: universal reverse primer |
| GAPDH | Forward: 5'-GCACCGTCAAGGCTGAGAAC-3' |
|  | Reverse: 5'-TGGTGAAGACGCCAGTGGA-3' |

Note: RT-qPCR, reverse transcription quantitative polymerase chain reaction; NEAT1, nuclear-enriched abundant transcript 1; SNAI1, snail family transcriptional repressor 1; SOCS3, suppressor of cytokine signalling-3; 4-Oct, 4-octamer-binding transcription factor; GAPDH, glyceraldehyde-3-phosphate dehydrogenase.
